# Effect of Preconception Care on Anemia and Body Mass Index among Women in a Rural and Tribal Area, Nashik District, India

**DOI:** 10.1177/26884844251379357

**Published:** 2025-09-26

**Authors:** Prakash Doke, Jayashree Gothankar, Amruta Chutke, Sonali Palkar, Prasad Pore, Rupesh Deshmukh, Archana Patil, Aniruddha Deshpande, Khanindra Bhuyan, Madhusudan Karnataki, Aparna Shrotri, Ravindra Chaudhari, Mohan Bacchav, Motilal Patil

**Affiliations:** ^1^ Department of Community Medicine, Bharati Vidyapeeth (DTU) Medical College, Pune, Maharashtra, India.; ^2^ Health Services, State Family Welfare Bureau, Pune, Maharashtra, India.; ^3^ UNICEF, Maharashtra, India.; ^4^ RCH Officer Zilla Parishad, Health Department, Nashik, Maharashtra, India.; ^5^ Taluka Health Officer Panchayat Samiti Office, Health Department, Nashik, Maharashtra, India.

**Keywords:** anemia, BCC, BMI, preconception care, primary health care, tribal block

## Abstract

**Objective:**

Preconception care reduces adverse maternal and neonatal outcomes. Despite the World Health Organization’s recommendations, it has not been uniformly implemented. The specific objectives of the study were to measure the change in calorie and protein intake, body mass index (BMI), and hemoglobin among women receiving preconception care, including behavioral change communication (BCC).

**Materials and Methods:**

We conducted an implementation study with the government’s support. Accredited social health activists conducted a house-to-house survey in four blocks (2 for intervention and 2 for comparisons). Each arm had one tribal and one rural block. They enrolled women desiring pregnancy within a year and collected desired information, including a 24-hours dietary recall. A repeat diet survey was carried out after 6 months. The authors provided preconception care in the intervention blocks. Health care workers followed the women monthly. The study was conducted from 2018 to 2020.

**Results:**

The study enrolled 7875 women from four blocks. In the intervention group, the proportion of women with very low-calorie intake reduced from 1.1% to 0.1%, and very low-protein intake reduced from 0.9% to 0.2%. In the comparison group, very-low-calorie intake was reduced from 1.7% to 1.5%, whereas very low protein intake was reduced from 1.6% to 0.8%. The proportion of underweight (BMI < 18.5) decreased from 40.70% at enrolment to 15.35% at the last follow-up. The mean hemoglobin improved from 10.56 gm% (SD = 1.25) to 11.10 gm% (SD = 1.07).

**Conclusions:**

The provision of preconception care, including BCC activities, through the public health system optimized the BMI and increased hemoglobin.

## Introduction

The first evidence of the benefits of preconception health promotion dates from 1987.^1^ The United States Centers for Disease Control was the first to recommend preconception health in 2006.^2^ Preconception care includes risk prevention, mitigation, and health promotion to improve health outcomes for mothers and children.^3^ The Government of India also focuses on planning pregnancy and provides guidelines to women about healthy behavior and practices before conception to ensure the best health outcome.^4^

India has the highest number of babies with low birth weight (LBW) globally.^5^ It is the most critical determinant of neonatal mortality. Anemia, grand multiparity, chronic medical illness, and non-consumption of iron folic acid (IFA) are associated with LBW.^6,7^ Higher education, optimal age (20–35 years), normal weight, and (IFA consumption are protective factors.^6,8^ A woman’s body mass index (BMI) is a major risk factor for childhood undernutrition in India.^9^ Despite Maharashtra being one of the prosperous states in India, as high as 54.5% of women between 15 and 49 years of age are anemic, 20.8% have low BMI (<18.5 kg/m^2^), and 23.4% have high BMI (>25 kg/m^2^) in 2019–2020.^10^ Women’s health before conception is the mediator for better newborn outcomes. World Health Organization’s (WHO’s) guidelines identify risk factors and emphasize the need to address them. Most of them are women-related.^11^ Among the risk factors, some are modifiable and some are not.

UNICEF supported the Government of Maharashtra and the authors’ medical college for implementing the preconception care project in four blocks (2 blocks for intervention and 2 for comparison) to reduce adverse pregnancy outcomes. The results showed that in the study arm, 11.18% were preterm births; in the comparison arm, 14.99% were preterm births; the proportion of LBW babies was 9.23% and 11.25%, respectively.^12^ Nutritional supplements during preconception have been shown to benefit the nutritional status of children born to women.^13^ However, studies showing the effects of the intervention on women’s health before conception are rare. Notably, without food supplementation during the preconception period, improvement in women’s health is unknown. Despite knowing that preconception care is a gap in the continuity of services during the reproductive age group of women and its effect on the health of the women and, thereby, the pregnancy outcome, it is not uniformly implemented. Women from tribal areas are known to be vulnerable. The National Family Health Survey (NFHS) does not mention tribal women. We selected feasible and relevant preconception care interventions similar to WHO recommendations for improving women’s health before conception.^11^

## Objectives

(1) To measure the change in hemoglobin (Hb) level and BMI in the intervention arm;
(2) To measure the change in the proportion of optimal health for planning a pregnancy in the intervention arm;
(3) To compare the change in calorie and protein intake among women after receiving preconception health care, including behavioral change communication (BCC);
(4) To compare the changes in calorie and protein intake among women in the intervention and comparison arms.

## Material and Methods

### Study design

It was a community-based implementation study and involved a comparison area.

### Setting

The authors conducted the study in the Nashik district of Maharashtra state. As per the last census, the district’s population is 6,107,187, of which 57.5% is rural (including 25.6% tribal). Out of 15 blocks in the district, 9 are tribal. The district has 106 primary health centers (including 52 tribal). The authors randomly selected one tribal (Peint) and one non-tribal (Sinnar) block for intervention and one adjacent tribal (Trimbakeshwar) and one non-tribal block (Niphad) for comparison. The selected intervention blocks had 13 primary health centers providing services to a population of 400,929 scattered in 273 villages. The population of comparison blocks was 726,973, scattered in 266 villages covered by 15 Primary Health Care (PHCs). The infrastructure and manpower in both areas were as per the population norm and almost similar. The government notifies administrative blocks that have a substantial population of tribes as tribal. Usually, the tribal communities reside in forests and hilly areas and are not very keen to join the mainstream.

### Study population and selection of participants

In the baseline house-to-house survey, all women in the reproductive age group (15–45 years) residing in the villages of the selected four blocks were included.

#### Inclusion criteria

A married woman resident of the selected block, desiring to conceive in the succeeding year.

#### Exclusion criteria

(1) A woman not knowing any language from Marathi, Hindi, or English;
(2) A critically ill woman;
(3) A woman who is unable to respond due to psychiatric problems.

### Interventions

The authors executed most of the relevant interventions recommended by WHO.^11^ Out of 13 groups, interventions related to genetic investigations plus counseling, interpersonal violence, mental health, psychoactive substance use, vaccination, and female genital mutilation were not considered, as the project was implemented through the primary level of health care. The interventions included an initial clinical examination and necessary laboratory investigations. Treatment, counseling, and referrals were provided for diagnosed conditions. Other interventions included 6-monthly deworming, anemia treatment/prevention, and daily folic acid supplementation during the pre-pregnancy phase. The study offered temporary family planning methods for women in the adolescent age group, those having severe anemia, short inter-pregnancy intervals, and/or low BMI. BCC included information about all the above points, as well as about quitting alcohol and tobacco, safe abortion centers, and BMI normalization.^12^ Women from the comparison area were free to approach whenever they desired. We did not undertake preconception interventions for women in the comparison area.

### Behavioral change communication

BCC components included all the above services plus nutrition for all women. Women having a BMI <18.5 or >25 received personal counseling for normalization of BMI. Three rounds of BCC were undertaken for all women. Accredited social health activists (ASHAs) and auxiliary nurse midwives (ANMs) used flipbooks to conduct the BCC session during the first round. The flipbook consisted of information related to preconception care. ASHA is a woman volunteer from the community and serves as a bridge between the government and the community. Her activities are primarily focused on maternal and child health. ANM is a formally trained nurse posted at a subcenter. During the second visit, a documentary containing preconception care information was shown to them. Twelve text and voice messages were sent on their mobile phone over 6 weeks in the third round. The authors gave specific diet charts to women with low or high BMI. ANM gave all these services with the help of ASHA. Medical officer (MO) and the lady health visitor (LHV) monitored the activities and checked the data quality. LHV is the regular supervisor for the activities conducted at the village and subcenter levels.

### Study tools

The authors prepared data collection tools for enrolling and following up with the women. The study team designed the BCC material. All these tools were in the local language (*Marathi*). All the tools and BCC materials were validated and pretested.

### Study period

The study started on April 7, 2018 with a kick-off meeting of all stakeholders. ASHAs enrolled most women by July 2018, depending on the population and workload. The monthly follow-up of the women for preconception care, including BCC, began in August 2018 and continued until October 31, 2020 (27 sessions covering the post-COVID-19 period after April 2020).

### Collection of data and monitoring

The authors trained all cadres of health care providers about the project activities and data collection. An application software for data entry and monitoring was created and installed on tablets provided to ANMs. ASHAs conducted the first house-to-house survey to enroll women desiring pregnancy in the subsequent year and collect basic information. Physical assessment of enrolled women was done by measuring height and weight using standard equipment and manner at home, or *Anganwadi*, or the nearest health facility, that is, subcenter or PHC. ASHAs recorded height to the nearest 0.1 cm and weights to the nearest 0.1 kg with standard precautions. They also conducted a diet survey using a 24-hours dietary recall method. A qualified and experienced nutritionist from our institution estimated participant women’s daily calorie and protein intake from the data. The research team, block facilitators, LHVs, and ANMs supervised the survey. ASHA brought all the enrolled women to the monthly village health, sanitation, and nutrition day (VHSND) till they conceived (confirmation by urine pregnancy test). In a few large villages, days other than VHSND were inevitable in providing adequate attention to the enrolled women. The MO at the PHC did the first check-ups. Later check-ups and services were provided by MO or ANM. After pregnancy detection, the woman received routine antenatal care services. ANM submitted monthly monitoring information using the software application. Camps were organized at the village or subcenter level to collect blood samples for laboratory investigations of the enrolled women.

All women who did not become pregnant were followed up every month to assess their health status, including weight. Hb was estimated during every quarterly session (1st, 4th, 7th, 10th, and so on). The baseline and fourth session Hb data (i.e., the immediate next opportunity of estimation) of the women whose Hb estimation was not done at the first session were combined. After the emergence of the COVID-19 pandemic, the government stopped Hb estimation during VHSND. The ANMs estimated Hb using Sahli’s method. BCC and other services were provided on the VHSND. As stated in the interventions, ANMs provided IFA or FA and deworming tablets following the government’s guidelines. The MO and ANM also inquired about Reproductive tract infection/scheduled Tribe (RTI/STI) symptoms or other signs and symptoms and provided appropriate counseling/treatment/referral. She maintained a record of all the activities conducted.

The study repeated the diet survey in all four blocks 6 months after enrolment, regardless of the participants’ pregnancy status, and again estimated their intake against the recommended daily allowance (RDA).

The authors used the WHO definition of anemia and BMI for classification.^14,15^

*Optimal health for the planning of pregnancy* was defined as “A woman whose age is between 20 to 30 years, BMI is 18.5 to 24.9 kg/m^2^, whose hemoglobin level is 12 gm% or more, is nulliparous or first para or second para, with the inter-pregnancy interval more than two years.” The definition evolved from NHM’s advice for pregnancy planning.^4^ It was validated by professors from the community medicine and obstetrics/gynecology departments and a subject consultant from UNICEF. At any time during the preconception period, a 10% increase in weight among underweight individuals, a 10% decrease in weight among overweight individuals, and more than a 10% rise in Hb among anemic women were labeled desired changes.

The study defined the nuclear family as parents living with their unmarried children, and other families as non-nuclear.

### Sample size and sampling

As mentioned under the study design heading, the primary study assessing the effect of intervention on adverse pregnancy outcomes estimated a sample size of 1374 women in each arm.^12^ However, all women desiring pregnancy in the succeeding year and willing to participate in the four blocks were enrolled, so the number of women studied was more than the required sample size.

### Data analysis

We combined severe and moderate anemia (<11 gm%). For categorical variables, data are presented as proportions and percentages. For continuous variables, the mean and standard deviation (SD) are calculated. The data presented in the figures were analyzed using the number of women attending that particular scheduled day-session and their BMI and Hb. We calculated the difference between BMI at baseline (including within a month) and the last follow-up visit. The change in BMI was tested for linear trend by applying the Chi square test. A similar process was adopted to measure the difference in Hb. The authors used a *t-*test to determine the calorie and protein intake differences. We dichotomized the findings (as underweight versus others, normal weight versus others, overweight versus others, or Hb less than 11 versus others, Hb 11–12 versus others, and Hb 12 or more versus others) in tables for comparison. We also used a mixed-method model to determine the association between intake and social factors that have shown a significant association at baseline. The level of significance was taken at *p* < 0.05.

### Ethical considerations

The Institutional Ethics Committee approved the study. It was registered under Clinical Trial Registry India (CTRI) vide CTRI registration number CTRI/2018/06/014657, dated June 28, 2018. We obtained written informed consent from all participants before inclusion in the study.

## Results

The investigators received excellent cooperation from all levels. All the expected personnel attended the kick-off meeting. The honorable health minister released the Dissemination Report in the presence of all senior officers and UNICEF consultants. There was initial resistance from ASHAs to perform the activities, but after some interactions and performance-based financial incentives, they fully cooperated with the study team. The women were made aware of the undesirable consequences of their health on the newborn. They accepted the counseling from ASHAs and shared and discussed their health problems. The diet survey results from four blocks are presented first, followed by the results of the intervention on women’s health in the two study blocks.

The study enrolled 7875 women desiring pregnancy. Missing data for any variable were less than 15%, except for the caste. The sociodemographic and dietary intake information is given in Table 1. The mean age of the women in the intervention group was 23.13 (SD = 3.76) years, whereas the mean age in the comparison group was 23.24 (SD = 3.67) years. Some demographic information about these women is already published.^16^

**Table 1.** Social and Diet Characteristics of Women, Nashik District, India, 2018–2020

	Group			Chi square value	*p*-value
	Intervention *N* = 3574^a^ (%)	Comparison *N* = 4301^a^ (%)	Total 7875^a^ (%)		
Age in years					
<20	602 (16.8)	654 (15.2)	1256	11.08	0.011
20–29	2756 (77.1)	3309 (76.9)	6065		
30–39	189 (5.3)	262 (6.1)	451		
40+	9 (0.3)	2 (0)	11		
Caste					
ST^b^	1493 (41.8)	1097 (25.5)	2590	142.11	<0.001
Non-ST	1635 (45.7)	2212 (51.4)	3847		
Type of family					
Nuclear	446 (12.5)	559 (13)	1005	1.14	0.28
Non-nuclear	2765 (77.4)	3220 (74.9)	5985		
Education					
<SSC^c^	1287 (36)	1553 (36.1)	2840	0.44	0.51
>SSC	2140 (59.9)	2502 (58.2)	4642		
Working					
Employed	1027 (28.7)	1666 (38.7)	2693	109.18	<0.001
Unemployed	2370 (66.3)	2301 (53.5)	4671		
Parity					
0	1753 (49)	1903 (44.2)	3656	19.66	<0.001
1	1141 (31.9)	1541 (35.8)	2682		
2	409 (11.4)	529 (12.3)	938		
3+	170 (4.8)	201 (4.7)	371		
Short stature^d^					
Yes	499 (14)	567 (13.2)	1066	0.31	0.58
No	2859 (80)	3371 (78.4)	6230		
BMI					
<17.0	1355 (37.9)	1400 (32.6)	2755	22.74	<0.001
<18.5	1584 (44.3)	1894 (44)	3478		
18.5 to 24.9	219 (6.1)	266 (6.2)	485		
≥25.0	133 (3.7)	223 (5.2)	356		
≥30.0	38 (1.1)	55 (1.3)	93		
Less calorie intake^e^					
Yes	40 (1.1)	71 (1.7)	111	3.95	0.047
No	3529 (98.7)	4228 (98.3)	7757		
Less protein intake^e^					
Yes	33 (0.9)	71 (1.7)	104	7.89	0.005
No	3535 (98.9)	4228 (98.3)	7763		

^a^ Totals do not tally due to non-responses.

^b^ Scheduled tribe.

^c^ Secondary school certificate (10 years of schooling).

^d^ Height less than145 cm.

^e^ Less than 50% of the recommended daily allowance.

### Dietary intake (four blocks)

The calorie and protein distributions were not normal (Kolmogorov–Smirnov). In both arms, after 6 months, there was an improvement. However, the magnitude of change was far higher in the intervention group (Table 2). The difference the increase in protein intake in the intervention group was more than double the increase in the comparison group. Similarly, the difference the increase in calorie intake in the intervention group was more than one and a half times the increase in the comparison group. The initial calorie intake of less than 50% in the intervention group was reduced by 87.5%, whereas in the comparison group, it was reduced by 7.04%. The protein intake of less than 50% was reduced by 75.76% in the intervention group and 47.89% in the comparison group. We ran a mixed-method model with fixed effects and observed that calorie intake is not associated with age, stature, BMI, or caste but with women’s occupation (*F* = 6.17; *p* = 0.013). However, protein intake was not associated with any of the studied variables.

**Table 2.** Diet Surveys in Nashik District, India, 2018–2020

	Calories				Proteins			
	Intervention		Comparison		Intervention		Comparison	
	First survey women (%)	Repeat survey women (%)	First survey women (%)	Repeat survey women (%)	First survey women (%)	Repeat survey women (%)	First survey women (%)	Repeat survey women (%)
% Intake of RDA								
0–24	0 (0.0)	0 (0.0)	0 (0.0)	0 (0.0)	0 (0.0)	1 (0.02)	2 (0.05)	2 (0.04)
25–49	40 (1.1)	5 (0.1)	71 (1.7)	66 (1.5)	33 (0.9)	7 (0.2)	69 (1.6)	35 (0.8)
50–74	2551 (71.5)	752 (21.1)	2623 (61.0)	1563 (36.3)	1965 (55.1)	451 (12.6)	2274 (52.9)	1028 (23.9)
74–99	933 (26.1)	2363 (66.2)	1518 (35.3)	2360 (54.9)	1354 (37.9)	1660 (46.4)	1653 (38.5)	1922 (44.7)
100+	45 (1.3)	450 (12.6)	87 (2.0)	312 (7.3)	216 (6.1)	1451 (40.6)	301 (7.0)	1314 (30.6)
Total	3569	3570	4299	4301	3568	3570	4299	4301
Median intake (IQR)	1593 (1432–1790)	1980 (1795–2201)	1665 (1495–1876)	1880 (1614–2101)	40.00 (36.20–45.90)	52.00 (45.00–60.00)	40.40 (36.00–46.30)	50.60 (41.30–56.45)
Mean intake (SD)	1631.34 (262.50)	1998.44 (301.72)	1696.48 (287.68)	1864.48 (343.84)	41.62 (7.78)	52.94 (7.78)	41.74 (8.19)	49.54 (10.64)
*t* test	65.48; *p* < 0.001		29.66; *p* < 0.001		59.52; *p* < 0.001		45.30; *p* < 0.001	

IQR, interquartile range; SD, standard deviation.

### Other variables intervention group (two blocks)

The number of women for monthly follow-up visits substantially reduced after April 2020 (emergence of COVID-19). Initially, we thought of giving results up to April 2020; however, we later decided to provide all results, giving clear information until the end of the project period. The overall mean number of follow-up visits was 9.47 (SD = 6.89), for women who became pregnant during follow-up was 7.18 (SD = 5.33), and 12.17 (SD = 7.52) for those who did not become pregnant.

A total of 182 women (2.30%) did not attend any preconception care sessions. Most of these women were from the non-tribal block of Sinnar (148 women, 81.32%). By default, for these women, the first and last findings/measurements were the same.

During the study, 1941 women became pregnant at various follow-up visits in the intervention arm.

### Body mass index

**FIG. 1.**
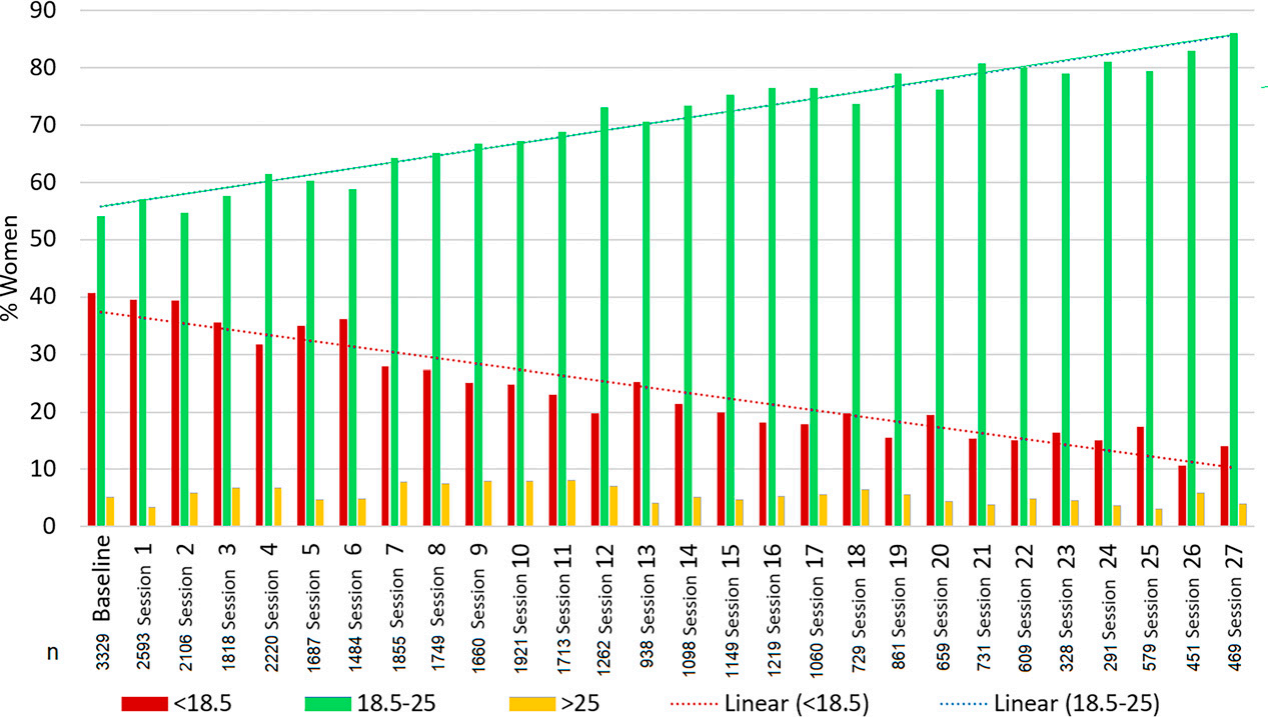
Change in BMI among enrolled women in the blocks from baseline to last follow-up visit, Nashik district, India, 2018–2020. BMI, body mass index.

Among the enrolled women, 12.86% were short-statured. Figure 1 depicts the number of women attending the scheduled day sessions and their category-wise BMI. At baseline, the mean BMI was 19.45 kg/m^2^ (SD = 3.03), which changed to 20.79 kg/m^2^ (SD =2.36) at the last, that is, 27th session (women who did not become pregnant and attended the last, i.e., 27th visit). The proportion of underweight (BMI <18.5 kg/m^2^) decreased from 40.7% at enrolment to 15.35% at the last session, and the proportion of overweight (BMI >25 kg/m^2^) decreased from 5.14% to 4.05% and the proportion of normal weight (BMI, 18.5–25 kg/m^2^) increased from 54.16% to 80.6%. The proportion of undernourished women decreased with increasing visits (chi square for trend = 1243.48, *p* < 0.001). Table 3 shows the change in BMI in all the enrolled women through paired analysis.

**Table 3.** BMI among Women Desiring Pregnancy in Intervention Area in Nashik District, India, 2018–2020

Initial classification	*N*	Before intervention mean (SD)	After intervention mean (SD)	*t*-value	*p*-value
Underweight BMI <18.5	1446	16.77 (1.24)	18.96 (2.63)	31.66	<0.001
Normal BMI 18.5–24.99	1978	20.78 (1.65)	20.97 (2.53)	3.46	0.001
Overweight BMI ≥25	150	27.73 (2.63)	25.13 (4.85)	7.02	<0.001

BMI, body mass index.

### Hemoglobin

**FIG. 2.**
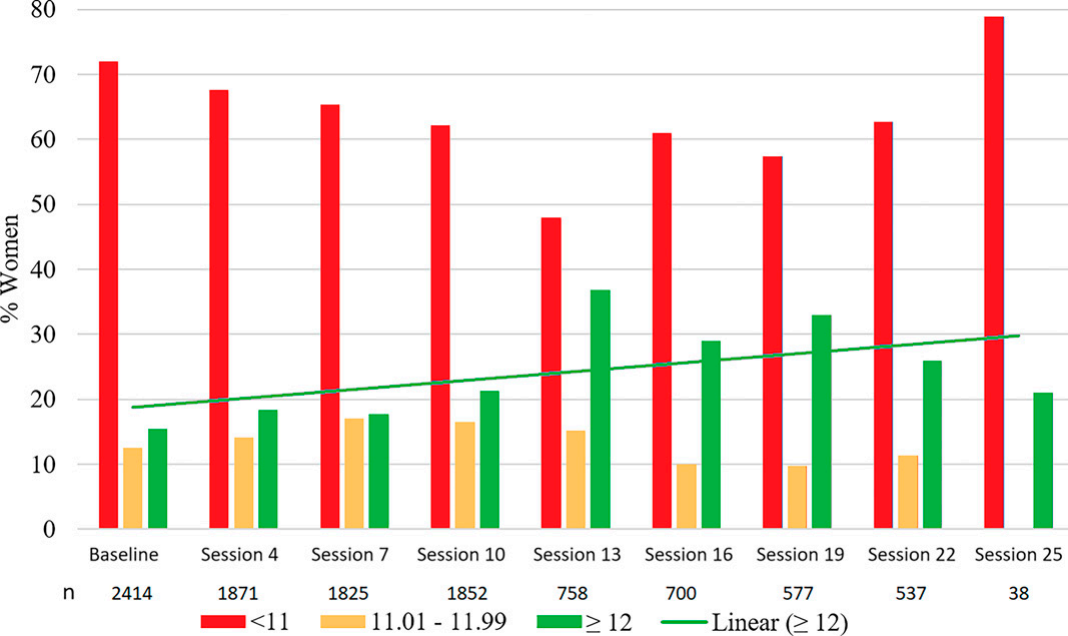
Change in hemoglobin levels (quarterly) among women in the blocks from baseline to last quarter (quarter 8th visit) follow-up, Nashik district, India, 2018–2020.

The study estimated Hb levels of 2414 women at the baseline and 154 women for the first time in the fourth session (those who did come in the first visit and did not conceive till then). The Hb was less than 12 gm% among 2168 out of 2568 women (84.42%), of which severe anemia (<8 gm%) was present among 1.01% of women, moderate anemia (8–11 gm%) in 70.17% of women, and mild anemia (11.01–11.99 gm%) among 13.24% women at baseline. In the tribal block, the proportion of anemic women was 87.91%, and in the non-tribal block, it was 80.72% before intervention. Figure 2 gives information about changes in anemia from baseline to 27 months of follow-up among women who attended the scheduled visits. At baseline, the mean Hb was 10.56 ± 1.25 gm%, which changed to 11.10 ± 1.07 gm% in the 22nd session. There was a slight reduction in the mean Hb at the 25th visit (10.28 ± 1.35 gm%).

The proportion of women with moderate to severe anemia decreased to 47.37% by the 13th session. But there was a slight increase later. The proportion of women with normal Hb (i.e., >12 gm%) had increased from 15.45% to 37% up to the 13th session. The proportion of non-anemic women increased as the visits increased (Chi square for trend = 21.88, *p* < 0.001).

Table 4 shows the mean Hb levels before and after intervention. The study estimated the Hb. of 72% of women before and 85% after the intervention.

**Table 4.** Hemoglobin Level among Women Desiring Pregnancy in Intervention Area in Nashik District, India, 2018–2020

Initial classification	*N*	Before intervention mean (SD)	After intervention mean (SD)	*t*-value	*p*-value
Moderate to severe anemia: Hemoglobin <11	1828	9.98 (0.81)	10.45 (1.12)	19.91	<0.001
Mild anemia: Hemoglobin 11.01–11.99	340	11.47 (0.22)	11.43 (0.80)	0.94	0.35
No anemia: Hemoglobin ≥12	400	12.57 (0.78)	12.03 (1.07)	9.73	<0.001

SD, standard deviation.

Application of the Chi square test revealed no association between sociodemographic variables (age, education, occupation, and parity) and desired changes in BMI among undernourished women. However, the desired change in Hb levels among anemic women was associated with age. Hb changes occurred in a higher proportion among women aged more than 23 years.

### Optimal health

At enrolment, 126 (3.53%; standard error of proportion = 0.31) women had optimal health, essential to planning a pregnancy. Later, the proportion of women with optimal health increased to 338 (9.46%; Standard error of proportion = 0.49; *p* < 0.05) at the last follow-up in non-pregnant women.

### Financial implications

The total direct cost of the project was about ₹ 10,000,000 ($120,000). Most of the direct cost was related to app development, preparation of video clips, publication charges, allowances to investigators, incentives to ASHAs, etc. In that order, indirect costs were minimal. We preponed most activities recommended during pregnancy.

## Discussion

Despite the COVID-19 pandemic, the study showed that preconception care, including the BCC, improved BMI and Hb levels. Folic acid supplementation continued until the end of the study period, as a lower dose was safe. After BCC sessions, which included dietary advice, the proportion of women consuming very few calories and proteins decreased. The proportion of women having optimal health significantly improved after interventions.

Poor maternal nutrition, maternal underweight, and maternal anemia are the risk factors for LBW.^17^ In low-income countries, 25% of LBW are attributable to maternal anemia during pregnancy,^18^ chronic hypertension, preeclampsia/eclampsia,^19^ and maternal age are other contributory factors. Infants born to women at extremes of reproductive age are at greater risk for stillbirth, preterm birth, neonatal death, congenital anomaly, and LBW.^20^

Pre-pregnancy underweight and overweight are associated with increased risks for adverse perinatal outcomes, including LBW.^21,22^ Pre-pregnancy BMI is a better predictor of LBW and preterm babies than weight gain during pregnancy.^23,24^ The low BMI in the preconception period is one of the most critical determinants of LBW and small-for-gestation babies.^25,26^ In the present study, about one-third of women were undernourished (BMI <18.5), similar to one study.^27^ We observed a higher proportion of underweight women than in the national-level district survey.^28^ The most likely reason may be the non-inclusion of the urban regions in the present study. In one study from China, 16.3% of the women were underweight, and 12.3% were categorized as overweight before pregnancy.^22^ Our finding contrasts the NFHS statistics of the last two rounds of the Nasik district (2015–2016 and 2019–2020). The proportion of underweight women in the reproductive age group with low BMI hovers around 25%.^28^ While interacting with participants, women informed us that they are now taking one additional meal, which may be the reason for their improvement.

The results of Hb estimation by different methods are not unequivocal. Although Sahli’s method has wider variation,^29–31^ agrees better with the autoanalyzer.^32^ But there may be arguments.^33,34^ Sahli’s method is used in the field. The present study revealed improvement in Hb levels during the preconception phase. However, the slight decline in non-anemic women after some visits in the present study may be due to difficulties in follow-up and provision of services during the COVID-19 pandemic. At enrollment in August, the harvesting season, about two-thirds of women came for Hb estimation; hence, the Hb estimation of non-pregnant women in November is combined with enrollment data. Less than 50% of preconception anemia observed in the present study was similar to the Tanzanian study.^35^ The latest estimate of anemia among non-pregnant women in the district was 55.4%.28. The high percentage of anemia in the present study is due to including a substantial number of tribal women. In Orissa also, a very high proportion of anemia (96.5%) was reported among adolescent girls.^36^ A meta-analysis observed that low maternal Hb during the preconception phase had the highest odds ratio for LBW, among all stages of the maternal cycle.^37^ The present study noted significant improvement in anemia, unlike in Vietnam.^38^ Despite implementing *Anemia Mukt Bharat*, the proportion of anemia among non-pregnant women in Maharashtra increased by 6.6% and by 4% in India.^10,39^ However, in the Nashik district, the proportion of non-pregnant anemic women slightly decreased from 55.4% to 54.6%.^28^ All reproductive-age women are expected to receive weekly prophylactic IFA supplementation; however, married women usually receive supplementation during pregnancy. We did not monitor prophylactic iron supplementation in the comparison area. Most pregnancies are unplanned, and therefore, starting preconception folic acid supplementation becomes ambiguous. Hence, the results of the present study are due to the interventions. The difference in Hb was small but significant. The total span of observed Hb is usually from 6 to 12 gm; hence, a large effect size is not expected. Few women deferred pregnancy till improvement in Hb level and BMI. A recent systematic review has shown no difference in pregnancy outcomes with and without multiple micronutrient supplementation during the preconception period.^40^ Only iron supplementation without BCC may not change the proportion of anemic women.^38^

The food intake is the root cause of anemia and undernutrition. Both calorie and protein intake were suboptimal. The sociodemographic factors were not associated with either calorie or protein intake, hence the changes are attributed to the interventions. In western India, the reported calorie intake was 2016 Kcal/day and 70.4 g of protein, which is far higher than we observed.^41^ In central India, among adolescent girls, intake of calories ranged from 69% to 86% of RDA, while protein intake was 71.5%–73.7% of RDA.^42^

The proportion of unintended pregnancies in India varies from 16% to 50% in various states.^43–45^ For planning pregnancy, the couple must have optimal health.

The Government of India has already advised most of the investigations, such as blood sugar, Human Immunodeficiency Virus (HIV), Thyroid stimulating Hormone (TSH), Venereal disease research laboratory (VDRL), and interventions, deworming, prophylaxis for anemia, which are executed during initial antenatal visits. In short, we recommend preponing these interventions in the preconception phase; therefore, financial implications are minimal. Encouraged by the results, the Government of Maharashtra vide Government Resolution No. Mis-2023/F.No.587/FW, Date: January 25, 2024, has included the preconception component in its comprehensive “*Vatsalya*” program.

It was a reasonably large study using the government primary-level system. The ASHAs receive performance-based payments for the implementation of various national program components. In the project, we also adopted a similar strategy. While implementing preconception care across the state, the government may decide on some performance-based incentives for ASHAs, similar to other programs. It was conducted in rural areas, including tribal areas. There were no monetary incentives or nutritional supplementation for the women. Our study may be the first interventional study demonstrating significant improvement in the proportion of women with low BMI, mainly through BCC interventions.

### Limitations

We could not maintain monthly follow-ups after April 2020 due to the COVID-19 pandemic. The supplies were irregular, women were afraid to attend services, and health workers were involved in control activities. The improvement in anemia may not be solely attributed to the BCC, as treatment/prophylactic IFA supplementation was given. There was a suboptimal focus on husbands and mothers-in-law. We did not focus on micronutrients. Women were irregular for follow-up, and migration, cultivation, harvesting, and festivals were the main hurdles. We did not differentiate between tribal women and those residing in tribal areas, as they share the same socio-environmental factors.

## Conclusions

Positive changes occurred without any financial incentives or nutritional supplements. The infrastructure, human resources, supplies, and guidelines exist. The existing government health system can deliver. Acceptance of our model in any area may reduce the proportion of underweight women before conception and, thereby, the prevalence of LBW.

## Recommendations

It is a prime need to move preconception care from research mode to service mode.

(1) Prepare standard guidelines and disseminate them for the universalization of preconception care;
(2) Train health care workers;
(3) Strong BCC components, including nutrition, need to be included. Frequent BCC cycles need to be carried out using different media. The inclusion of husbands and mothers-in-law will undoubtedly be beneficial. Interpersonal or group communication should be preferred. Mass media should also be used.
(4) Researchers and international organizations such as WHO and UNICEF may urge all policymakers, technocrats, bureaucrats, and people’s representatives to universalize preconception care.
(5) A robust system of monitoring needs to be developed.

## Abbreviations Used

ANMs: auxiliary nurse midwives
ASHAs: Accredited social health activists
BCC: behavioral change communication
BMI: Body Mass Index
CTRI: Clinical Trial Registry of India
Hb: Hemoglobin
HIV: Human Immunodeficiency Virus
IFA: Iron Folic Acid
LBW: Low birth weight
LHVs: Lady Health Volunteer
NFHS: National Family Health Survey
PHC: Primary Health Care
RTI: Reproductive tract infection
SD: Standard Deviation
SSC: Secondary School Certificate
ST: scheduled Tribe
STI: sexually transmitted infection
TSH: Thyroid stimulating Hormone
UNICEF: United Nations International Children’s Emergency Fund
VDRL: Venereal disease research laboratory
WHO: world health organization
